# sE-cadherin serves as a diagnostic and predictive parameter in prostate cancer patients

**DOI:** 10.1186/s13046-015-0161-6

**Published:** 2015-05-14

**Authors:** Igor Tsaur, Kristina Thurn, Eva Juengel, Kilian M. Gust, Hendrik Borgmann, Rene Mager, Georg Bartsch, Elsie Oppermann, Hanns Ackermann, Karen Nelson, Axel Haferkamp, Roman A. Blaheta

**Affiliations:** Department of Urology, Goethe-University, Frankfurt am Main, Germany; Department of Surgery, Goethe-University, Frankfurt am Main, Germany; Institute for Biostatistics, Goethe-University, Frankfurt am Main, Germany; Department of Vascular and Endovascular Surgery, Goethe-University, Frankfurt am Main, Germany

**Keywords:** Prostate cancer, Diagnosis, Prediction, Biomarker, sE-cadherin

## Abstract

**Background:**

Measurement of prostate-specific antigen (PSA) advanced the diagnostic and prognostic potential for prostate cancer (PCa). However, due to PSA’s lack of specificity, novel biomarkers are needed to improve risk assessment and ensure optimal personalized therapy. A set of protein molecules as potential biomarkers was therefore evaluated in serum of PCa patients.

**Methods:**

Serum samples from patients undergoing radical prostatectomy (RPE) for biopsy-proven PCa without neoadjuvant treatment were compared to serum samples from healthy subjects. Preliminary screening of 119 proteins in 10 PCa patients and 10 controls was carried out by the Proteome Profiler Antibody Array. Those markers showing distinct differences between patients and controls were then further evaluated by ELISA in the serum of 165 PCa patients and 19 controls. Uni- and multivariate as well as correlation analysis were performed to test the capability of these molecules to detect disease and predict pathological outcome.

**Results:**

Screening showed that soluble (s)E-cadherin, E-selectin, MMP2, MMP9, TIMP1, TIMP2, Galectin and Clusterin warranted further evaluation. sE-Cadherin, TIMP1, Galectin and Clusterin were significantly over- and MMP9 under-expressed in PCa compared to controls. The concentration of sE-cadherin, MMP2 and Clusterin correlated negatively and that of MMP9 and TIMP1 positively with the Gleason Sum at prostatectomy. Only sE-cadherin significantly correlated with the highest Gleason pattern. Compared to serum PSA, sE-cadherin provided an independent and better matching predictive ability for discriminating PCas with an upgrade at RPE and aggressive tumors with a Gleason Sum ≥7.

**Conclusions:**

sE-cadherin performed most favorably from a large panel of serum proteins in terms of diagnostic and predictive potential in curatively treatable PCa. sE-cadherin merits further investigation as a biomarker for PCa.

## Background

Prostate cancer (PCa) is currently the most prevalent male neoplasia in industrialized countries, accounting for almost 12 % of cancer cases annually [1]. Besides incumbent morbidity, the disease is associated with a significant economic burden. Declining mortality is expected to cause increases in healthcare costs as a result of increased diagnosis, diagnosis at an earlier stage and increased survival [2].

Due to the biological heterogeneity of PCa and rapidly expanding treatment options, individualized risk-adapted therapy incorporating patient- and tumor-specific characteristics is required to optimize outcome and avoid over-treatment with unnecessary adverse effects [3, 4]. Since not only radical prostatectomy (RPE) or radiation, but also active surveillance possibly postponing definite therapy, currently represent state-of-the-art care for patients with a localized PCa and a long life expectancy [5], identifying cancer types with high progression risk is indispensable in determining a treatment course. Nomograms, neural networks and predictive tables have all been developed in the past years to tailor decision-making at different stages of PCa diagnosis and treatment [6]. Being limited in their clinical application by problematic generalization and population-specific issues [7], they provide an average accuracy of 70 % [6]. To improve their performance, identification of appropriate molecular biomarkers and possibly their incorporation into predictive models is crucial [7].

Until now, prostate specific antigen (PSA) remains the only clinically relevant diagnostic and follow-up biomarker [8]. Recent research has been aimed at finding markers to overcome the limits of PSA, not only to diagnose PCa but also to distinguish between indolent and clinically significant disease [9]. Despite initial promising reports, these marker-candidates have fallen short following extensive validation and have not proven to be of routine value.

Marker detection in serum is attractive since sampling is simple and ideal for screening and prognostic evaluation of PCa to facilitate treatment choices for patients with local disease [10]. A panel of serum candidate markers known to be involved in inflammatory processes and tumorigenesis of different cancer types was evaluated for diagnostic and predictive potential in a cohort of patients with PCa.

## Methods

### Patients and controls

Study approval was granted by the local medical ethics committee (project number SUG_01-2014). Patients (n = 165) undergoing curative radical prostatectomy for biopsy-proven PCa without any neoadjuvant treatment between October 2010 and June 2014 in the Department of Urology, Goethe-University, Frankfurt am Main, Germany, were included in the study after having signed informed consent to utilize biomaterial for scientific assessment. The clinical tumor stage was classified according to the 7th edition of AJCC [11] and the pathological tumor stage was determined according to the 6th edition of the TNM classification [12]. Tumors were graded with the Gleason Sum (GS) [13]. Clinical and histological characteristics were collected from patient charts. Risk classification was determined according to D`Amico et al. [14]. Imaging was carried out in the high-risk group according to the guidelines of the European Association of Urology [15]. Epstein criteria [16] were used to assess the clinical significance of the tumor. Controls (n = 19) were healthy, age-matched, male volunteers.

### Blood samples

10 ml peripheral blood was drawn from patients several days before surgery and from controls. Blood samples were allowed to coagulate and then centrifuged at 3000 rpm at +4 °C for 10 min. The serum supernatant was stored at -80 °C.

### Proteome profiler antibody array

Serum from 10 PCa patients and from 10 controls were applied to a proteome profiler array to screen 119 markers (Human soluble Receptor Array Kit, Non Hematopoietic Panel, R&D systems, MN, USA) (Table 1) according to the manufacturer’s instructions. Briefly, membranes spotted with antibodies were blocked with Array buffer for 1 h at room temperature on a shaker followed by incubation with 1 ml serum mixed with 3 ml of array buffer overnight at 4 °C. On the next day membranes were washed 3x with wash buffer and further incubated with a detection antibody cocktail for 2 h at room temperature on a shaker. Membranes were again washed 3x with wash buffer and incubated with Streptavidin-HRP for 30 min at room temperature on a shaker. Signals were visualized using the Chemi reagent mix on a FUSION FX-7 imaging device (Vilber Lourmat, Torcy, France).

**Table 1:** Markers screened with Proteome Profiling Antibody

A: Non-Hematopoietic Array				
ADAM15	βIG-H3	BMPR-IB/ALK-6	Cadherin-4/R-Cadherin	Cadherin-11
Cadherin-13	E-Cadherin	N-Cadherin	P-Cadherin	VE-Cadherin
Cathepsin-D	CD40/TNFRSF5	CEACAM-5/CD66e	CHL-1/L1CAM-2	Clusterin
Coagulation-Factor II/Thrombin	COMP/Thrombospondin	CRELD2	Desmoglein 2	ECM-1
EGF R/ErbB1	Endoglycan	EpCAM/TROP-1	ErbB2/HER2	ErbB3/HER3
ErbB4/HER4	ESAM	Galectin-2	HPRG	Integrin α3/CD49c
Integrin α5/CD49e	Integrin α6/CD49f	Integrin α9	Integrin αV/CD51	Jagged 1
JAM-B/VE-JAM	JAM-C	LRP-6	MCAM/CD146	MEPE
MUCDHL	Nectin-2/CD112	Nectin-4	Neurotrimin	Notch-1
NrCAM	Periostin/OSF-2	Podocalyxin	E-Selectin/CD62e	Semaphorin-3A
SREC-I/SR-F1	SREC-II	Stanniocalcin 1	Syndecan-1/CD138	Syndecan-4
Thrombospondin-2	TIMP-4	TROP-2	VAP-1/AOC3	VCAM-1
VEGF R1/Flt-1	VEGF R2/KDR/Flk-1			
B: Common analytes array				
ACE	ADAM8	ADAM9	ADAM10	ALCAM/CD166
Amphiregulin	APP (pan)	BACE-1	BCAM	C1qR1/CD93
CD9	CD23/Fc ε RII	CD31/PECAM-1	CD36/SR-B3	CD40 Ligand/TNFSF5
CD44H	CD58/LFA-3	CD90/Thy1	CD99	CD155/PVR
CEACAM-1/CD66a	CX3CL1/Fractalkine	CXCL8/IL-8	EMMPRINN/CD147	Endoglin/CD105
Epiregulin	Galectin-1	Galectin-3	Galectin-3BP/MAC-2BP	HB-EGF
ICAM-2/CD102	IL-1 RII	IL-15 Rα	Integrin β1/CD29	Integrin β2/CD18
Integrin β3/CD61	Integrin β4/CD104	Integrin β5	Integrin β6	JAM-A
Lipoclain-2/NGAL	LOX-1/SR-E1	MD-1	MMP-2 (total)	NCAM-1/CD56
NCAM-L1	Osteopontin	PAR1	Pref-1/DLK-1/FA1	RECK
Stabilin-1	TACE/ADAM17	Thrombospondin	TIMP-1	TIMP-2
TIMP-3	TNF RII/TNFRSF1B			

### Elisa

E-selectin, soluble (s)E-Cadherin, MMP2, MMP9, TIMP1, TIMP2, Galectin and Clusterin, were identified as distinctly differently expressed in the serum of PCa patients and controls in the Proteome Profiler Antibody Array screening. The concentration of these markers was determined in the serum of 165 PCa patients and 19 controls using commercially available ELISA Kits (R&DSystems, MN, USA) according to the manufacturer’s instructions. Briefly, serum was diluted, pipetted into wells and incubated at 37 °C or RT for 1-2 h according to kit instructions. After incubation plates were washed 4x, followed by incubation with detection antibodies for 30 min or 1 h at RT and wells were subsequently washed 4x. TMB substrate was added to each well for 20 min at RT, followed by the addition of stop solution. The optical density was measured spectrophotometrically with an ELISA reader (Infinite M200 series, Tecan, Crailsheim, Germany). All assays were done in duplicate and the concentration was calculated from a standard curve using a 4-parameter curve fit (Magellan software, Tecan).

### Statistics

Univariate analysis was performed by the Wilcoxon-Man-Whitney-test for comparison between two groups and the Kruskal-Wallis-test with the Iman-Conover-method (Bonferroni-Holm-corrected) for more than two groups. Bi- and multivariate analysis was carried out by logistic regression for two target values and multiple regression for more than two target values. Correlation between two parameters was evaluated through Spearman’s coefficient. The statistical program applied was BiASfuer Windows (Version 9.11, Dr. rer. nat. Hanns Ackermann, epsilon-publishers, Frankfurt, Germany). The null hypothesis was rejected if p-values were less than 0.05. Continuous data were presented as median (range) or number (%), as applicable.

## Results

Clinical and pathologic demographics of 165 patients are shown in Table 2. The median age at tumor diagnosis was 65 years and the median serum PSA was 8.1 ng/ml. Nearly all PCas submitted to surgery were clinically significant. All patients were clinically free of visceral or bone disease. None of the patients had evident clinical signs of infection or acute or chronic inflammation at surgery. Histologically, all tumors were conventional acinar adenocarcinomas.

**Table 2:** Clinical and histopathological demographics of 165 PCa patients

	n = 165 (100 %)
Age, yrs	65 (range: 40-88)
pT-stage	
<3	113 (68.5)
≥3	52 (31.5)
Extracapsular infiltration	34 (20.6)
Infiltration of seminal vesicles	18 (11.0)
cT-stage	
<3	158 (95.8)
≥3	7 (4.2)
Serum-PSA, ng/ml	8.1 (1.8-136)
PSAD, ng/ml/ml	0.2 (0.1-1.8)
Abnormal DRE	65 (39.4)
Prostate volume, ml	33 (10-120)
Gleason Sum (Biopsy)	
≤6	86 (52.1)
7	55 (33.3)
≥8	24 (14.5)
Highest Gleason Pattern (Biopsy)	
3	70 (42.4)
4	62 (37.6)
5	14 (8.5)
Gleason Sum (RPE)	
6	27 (16.4)
7	99 (60.0)
≥8	39 (23.6)
Highest Gleason Pattern (RPE)	
3	28 (17.0)
4	104 (63.0)
5	33 (20.0)
Gleason Sum change	
upgrade	85 (51.5)
downgrade	15 (9.0)
Clinically significant PCa (Epstein)	158 (95.8)
D’Amico Classification	
Low risk	55 (33.3)
Intermediate risk	61 (37.0)
High risk	49 (29.7)
N+	22 (13.3)
R+	36 (21.8)
L+	29 (17.6)
V+	2 (1.2)
Pn+	106 (64.2)

Values expressed as median with range or number (%). *RPE*, radical prostatectomy; *DRE*, digital rectal examination, *PSAD*, PSA density

The screening analysis by Proteome Profiler showed distinctly altered expression of sE-cadherin, E-selectin, MMP2, MMP9, TIMP1, TIMP2, Galectin and Clusterin in the serum of tumor patients compared to controls. Further ELISA investigations were based on these markers.

**Fig. 1:**
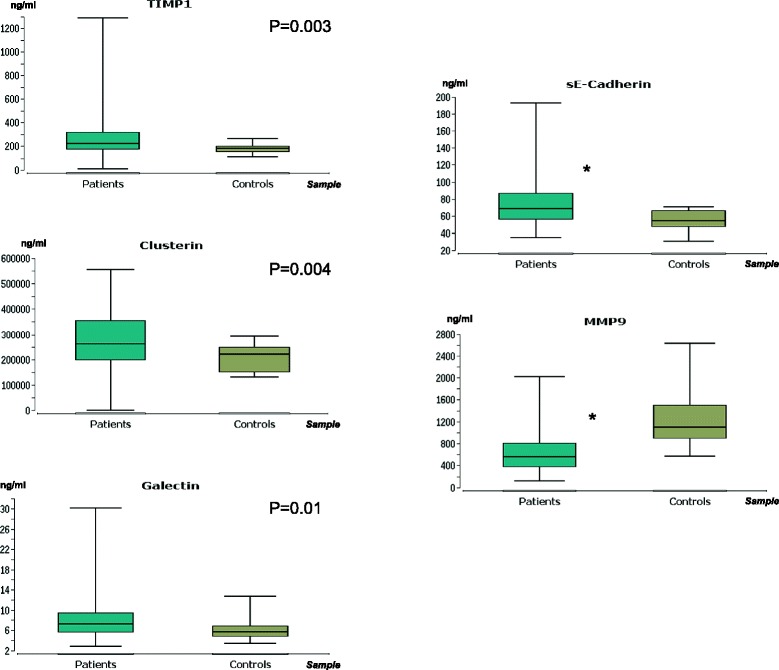
Marker concentrations in serum from tumor patients and controls. Only candidate molecules with significant differences are presented. Y-axis - concentration in ng/ml; X-axis - serum samples of tumor patients and controls. Box: lower line - quartile Q1 (25 %-quantile); middle line - median; upper line - quartile Q3 (75 %-quantile); aerials - extreme values. * - p < 0.001 (Wilcoxon-Mann–Whitney-test). 0.05 > p ≥ 0.01 are given in exact numbers

**Fig. 2:**
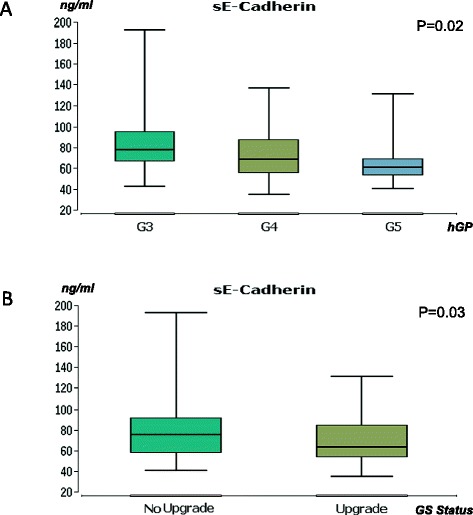
Associations between sE-cadherin and histopathological parameters. **a**: Association between sE-cadherin expression and highest Gleason pattern. Y-axis - concentration in ng/ml; X-axis - serum samples of tumor patients distributed by the highest Gleason pattern (hGP). G3-G5 - Gleason pattern 3-5. P-value (Kruskal-Wallis-test). **b**: Association between sE-cadherin expression and Gleason sum upgrade. Y-axis - concentration in ng/ml; X-axis - serum samples of tumor patients distributed by upgrade status of the Gleason sum in prostatectomy specimen. P-value (Wilcoxon-Mann–Whitney-test). Box: lower line - quartile Q1 (25 %-quantile); middle line - median; upper line - quartile Q3 (75 %-quantile); aerials - extreme values

Univariate analysis of the ELISA investigation demonstrated that serum sE-Cadherin, TIMP1, Galectin and Clusterin were significantly over- and MMP9 under-expressed in PCa patients compared to healthy controls (all p < 0.05, Fig. 1). The concentration of sE-Cadherin, MMP2 and Clusterin correlated negatively and that of MMP9 and TIMP1 positively with GS at prostatectomy (all p < 0.05, Table 3), but only sE-cadherin significantly correlated with the highest Gleason pattern (p < 0.05, Fig. 2a). In the group of patients with a GS upgrade from prostate biopsy to prostatectomy specimen, a decreased concentration of sE-cadherin was observed, compared to patients without upgrade (p < 0.05, Fig. 2b).

**Table 3:** Correlation of markers with prostatectomy Gleason Sum

	sE-Cadherin	MMP2	MMP9	TIMP1	Clusterin
p-value	0.004599	0.020268	0.048087	0.027881	0.031574
Coefficient (rho)	−0.219991	−0.180763	0.154655	0.171867	−0.168062

Multivariate analysis with logistic regression for all 8 candidate markers as well as serum PSA was unsuitable because, taking into account the cohort size and ‘the rule of ten’ [17], sufficient stability of the model could not be ensured. Therefore, a bivariate logistic regression analysis was performed, incorporating each of the candidate molecules and serum PSA to assess their ancillary predictive potential over PSA for clinical and pathological T-stage, L-, N-, R-, Pn-status, biopsy (b) and prostatectomy (p)GS after dichotomization of the study cohort into GS < 7 and ≥ 7, as well as Gleason upgrade. Only sE-cadherin yielded favorable discriminative ability over PSA for more aggressive tumors with a pGS ≥ 7 (p = 0.01). Moreover, sE-cadherin better detected PCas with a Gleason upgrade at RPE (p = 0.01) than did serum PSA. Neither sE-cadherin, TIMP1, Galectin, Clusterin nor MMP9 correlated significantly with serum PSA.

## Discussion

Estimating PCa malignancy in an era of personalized healthcare requires novel diagnostic and prognostic biomarkers to develop individual treatment strategies. Therefore, the potential of a panel of serum proteins to detect PCa and predict histopathological outcome in curatively treated disease was evaluated.

Out of 119 candidate molecules, sE-cadherin yielded the most promising potential to serve as a biomarker in the current study cohort with almost 96 % clinically significant PCas according to the Epstein criteria [16]. E-cadherin, a type-I-cadherin, belongs to the cadherin family of transmembrane or membrane-associated glycoproteins located in the adherens junction and basolateral membrane in epithelial cells. It consists of a large extracellular domain, a transmembrane segment and a conserved cytoplasmic domain [18]. E-cadherin is indispensable in promoting cell-cell adhesion in a Ca^2+^-dependant manner and steers tissue morphogenesis [19]. During cancer progression, transcriptional E-cadherin reprogramming induces decreased adhesion and enhanced migration and invasion during the epithelial-to-mesenchymal transition of epithelial cells [20, 21]. sE-cadherin is an 80-kDa ectodomain of the full-length E-cadherin, which is proteolytically shed into the extracellular space through cleavage by secretases and caspases [18]. Concurrent to the disruption of adhesion junctions, EGFR and Wnt/β-catenin pathway signaling, often involved in tumorigenesis, are activated [22]. Since E-cadherin cleavage has been linked to neoplastic adenoma-cancer progression, serum levels of sE-cadherin have been shown to be augmented in patients with breast, gastric and colorectal cancer [23]. This is in line with our findings demonstrating that the serum concentration of sE-cadherin was elevated in patients with PCa, more than other investigated candidates. Thanks to its stability in blood, sE-cadherin may be a suitable indicator for early PCa detection, due to accumulation during tumor-associated proteolysis [23].

So far, little has been reported about sE-cadherin in connection with PCa. Ahmed et al. [24] investigated its potential as a biomarker in a group of 71 Egyptian males with PCa. In agreement with our findings, significant over-expression of sE-cadherin in the serum of tumor patients, compared to healthy individuals, was observed. No correlation with the clinical tumor stage was apparent. In contrast to our findings, the authors reported high expression of sE-cadherin, correlating with a poor Gleason grade and serum PSA. It is difficult to interpret their data in the context of our findings due to the limited sample size of that cohort, median PSA of 70.7 ng/ml for tumor patients, most cancers classified as stage D and details about their staging procedure absent. These study characteristics harbor significant probability of undetected metastasis and therefore deviate from the present study population, which was clinically free from visceral- and bone-disease. Furthermore, alterations in Gleason grading procedures over time have resulted in significant tumor up-grading [25], making a study published in 1999 [24] difficult to compare with the present study carried out more than a decade later, due to bias stemming from the Will Rogers phenomenon. Later, Kuefer et al. [26] observed no difference between the expression of serum sE-cadherin in 61 men with localized PCa compared to controls. On the contrary, a more contemporary series also involving 61 patients with clinically localized PCa, reported by Iacopino et al. [27], demonstrated serum sE-cadherin over-expression in PCa patients compared to that of healthy individuals, which is similar to our observation. However, no correlation of the marker with PSA, T-stage, GS, grade or R- and Pn-status was observed by those investigators. To best knowledge, the current assessment of sE-cadherin has taken place in the largest cohort of clinically non-metastatic males with PCa. sE-cadherin correlated with the highest Gleason pattern, which is an important prognosis factor [28] and was also associated with a Gleason upgrade and pGS, which is more accurate than bGS at predicting biochemical recurrence after RPE [29]. Most importantly, for identifying more aggressive tumors with GS ≥ 7 and tumors with a Gleason upgrade, sE-cadherin outmatched serum PSA without correlating with this marker.

The negative serum sE-cadherin correlation with more aggressive PCa in this investigation requires further research since serum sE-cadherin has been reported to rise with more aggressive or advanced stages in many other cancer types [23]. However, Juhasz et al. has provided evidence for significantly decreased expression of serum sE-cadherin in individuals with advanced and metastasized diffuse-type gastric cancer in contrast to intestinal cancer [30]. Careful analysis of cancer type as well as stage and grade specific molecular machinery pertaining to E-cadherin turnover is therefore warranted. Ongoing studies on sE-cadherin with respect to PCa should also take into consideration that proinflammatory cytokines [31], growth factors [32], as well as chemically induced oxidative stress [33] may contribute to its generation and possibly falsify results based on its expression.

Although sE-cadherin seems to be a promising biomarker, it may not be alone. Catenins, as well as CD44, are associated with E-cadherin in regulating prostate cancer cell adhesion [34]. In particular, CD44v8–10 have been shown to be negatively correlated with c-Myc expression, both in vitro and in vivo. The negative correlation may partly be attributable to redox stress-enhanced Wnt/beta-catenin signaling [35].

The reason no distinct difference was apparent between the CD44 serum level in healthy subjects and tumor patients may have been because CD44 variants such as CD44v5, CD44v6 and CD44v10, possibly involved in prostate cancer progression, were not individually evaluated [36,37]. EpCAM differences between tumor patients and controls were also not apparent in the present investigation, which is in line with other reports [38]. EpCAM has been reported to be responsible for persistence of minimal residual disease and latent relapse of prostate cancer, presumably by affecting the susceptibility to the EGF ligand and regulating the AMPK signaling pathway [39]. Nevertheless, a prognostic/diagnostic role of EpCAM in EGFR activated prostate cancer cells should not be ruled out.

The same ambivalence might apply to MMP9. Although this protein has been suggested to correlate with prostate cancer progression, the correlation has not always been substantiated. Recent experiments documented that MMP9 might even exert tumor-suppressive properties. In fact, elevated MMP9 secretion has been shown to result in decreased prostate cancer cell proliferation, coupled to increased sE-cadherin shedding [40]. The combined effect might explain the inverse correlation between sE-cadherin and MMP9 found in the present investigation, whereby reduced MMP9 and elevated sE-cadherin may drive tumor proliferation forward. Still, this is hypothetical and requires further evaluation.

The present investigation is limited by its single-center design and lack of external validation. To account for regional and racial disparity regarding the biological characteristics of PCa [41], validation studies with patient cohorts from other centers would be desirable. The potential of sE-cadherin as a biomarker could thereby be more exactly appraised.

## Conclusions

To sum up, sE-cadherin performed best out of a large panel of serum proteins in terms of diagnostic and predictive potential in 165 patients with clinically localized PCa. Therefore, this molecule merits further investigation as a biomarker for PCa.
